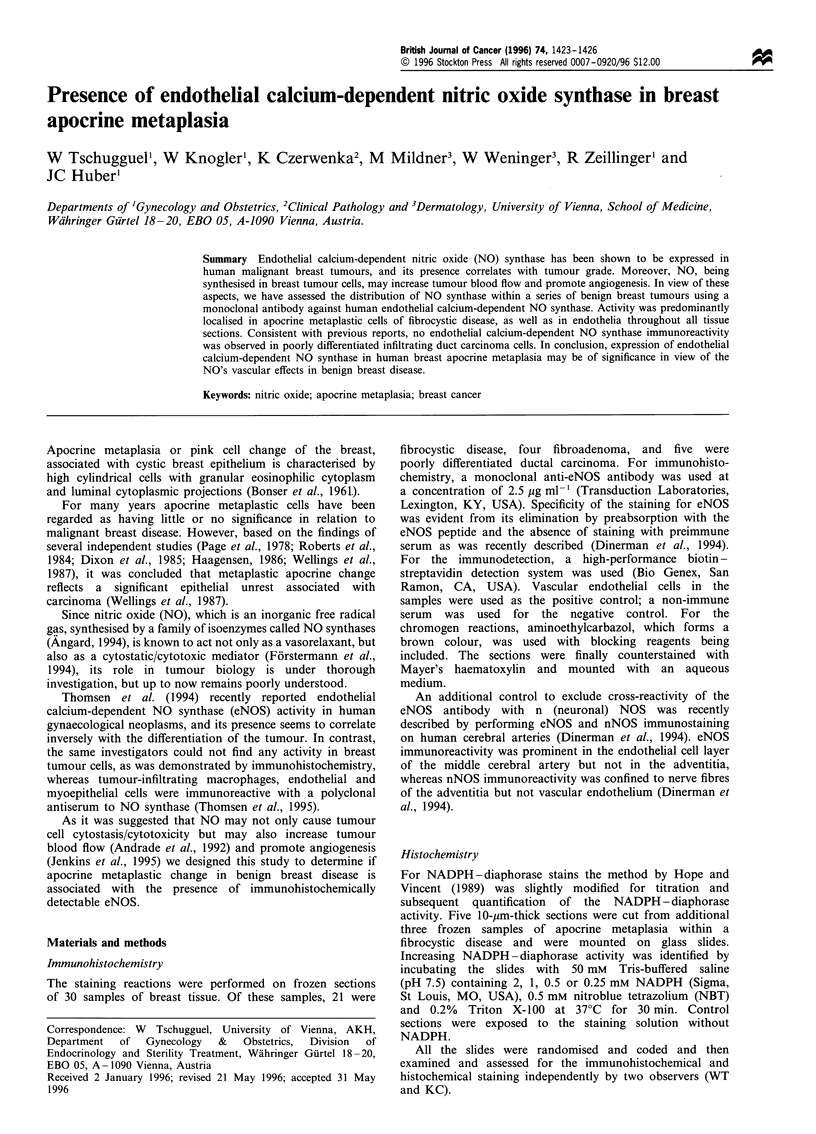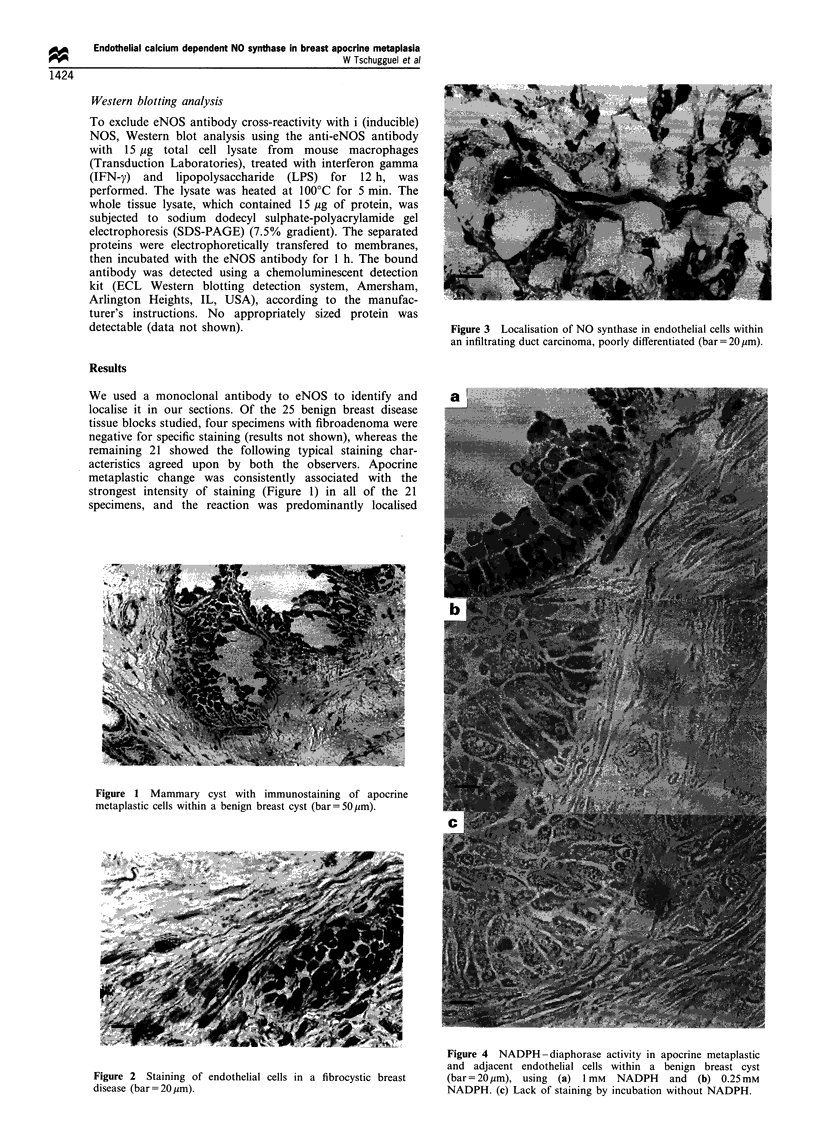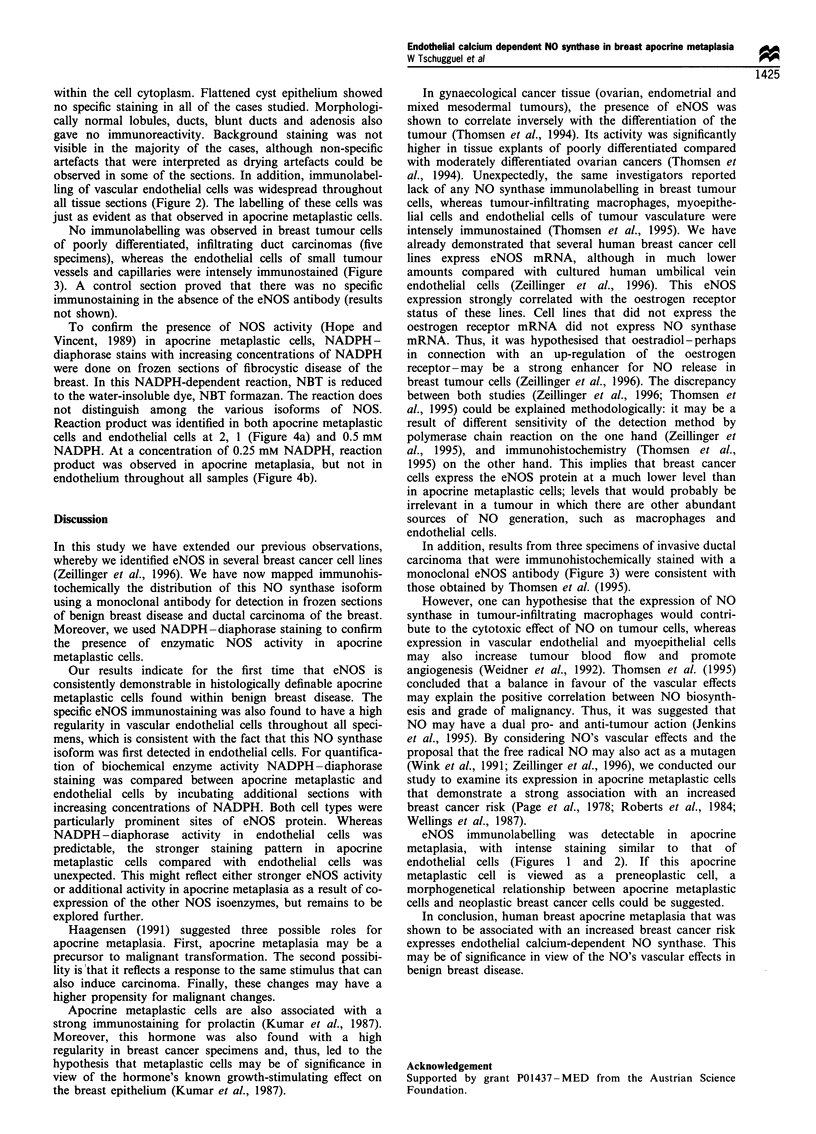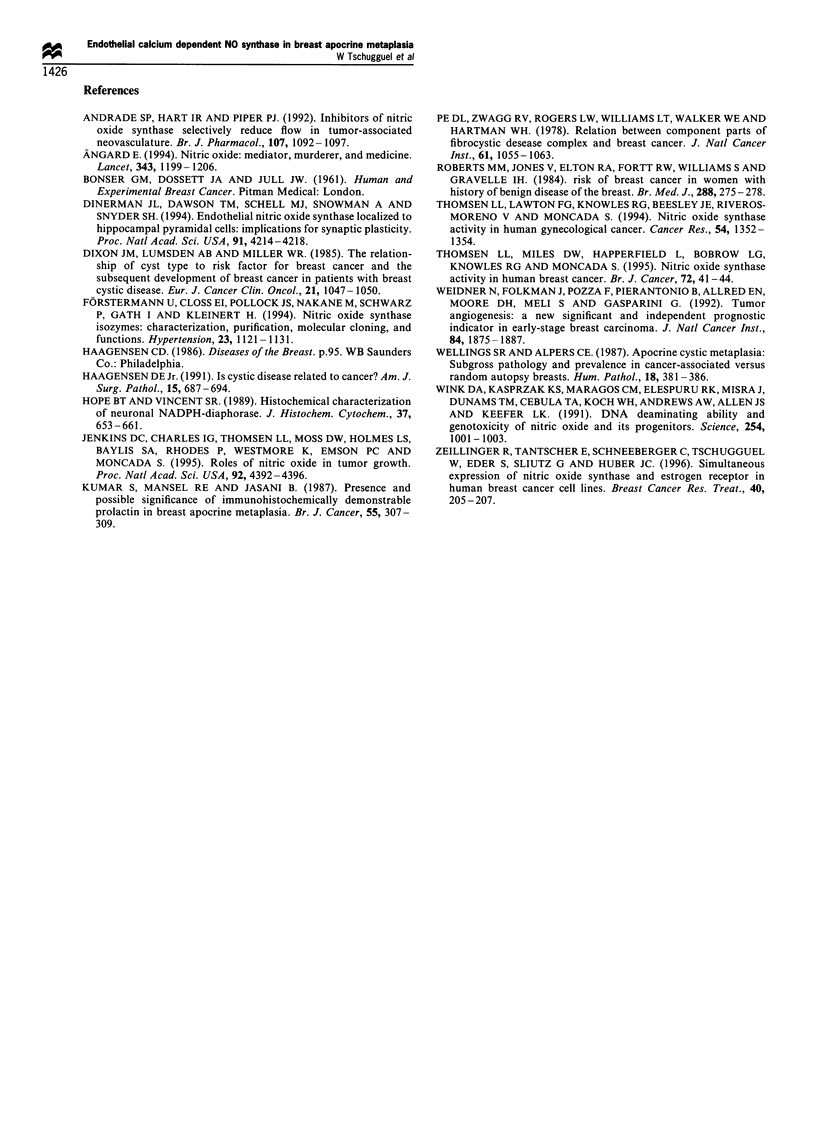# Presence of endothelial calcium-dependent nitric oxide synthase in breast apocrine metaplasia.

**DOI:** 10.1038/bjc.1996.559

**Published:** 1996-11

**Authors:** W. Tschugguel, W. Knogler, K. Czerwenka, M. Mildner, W. Weninger, R. Zeillinger, J. C. Huber

**Affiliations:** Department of Gynecology and Obstetrics, University of Vienna, School of Medicine, Austria.

## Abstract

**Images:**


British Journal of Cancer (1996) 74, 1423-1426
© 1996 Stockton Press All rights reserved 0007-0920/96 $12.00

### Presence of endothelial calcium-dependent nitric oxide synthase in breast apocrine metaplasia

W Tschugguel[1], W Knogler[1], K Czerwenka[2], M Mildner[3], W Weninger[3], R Zeillinger[1] and JC Huber[1]

*Departments of [1]Gynecology and Obstetrics, [2]Clinical Pathology and [3]Dermatology, University of Vienna, School of Medicine, Währinger Gürtel 18-20, EBO 05, A-1090 Vienna, Austria.*

**Summary** Endothelial calcium-dependent nitric oxide (NO) synthase has been shown to be expressed in human malignant breast tumours, and its presence correlates with tumour grade. Moreover, NO, being synthesised in breast tumour cells, may increase tumour blood flow and promote angiogenesis. In view of these aspects, we have assessed the distribution of NO synthase within a series of benign breast tumours using a monoclonal antibody against human endothelial calcium-dependent NO synthase. Activity was predominantly localised in apocrine metaplastic cells of fibrocystic disease, as well as in endothelia throughout all tissue sections. Consistent with previous reports, no endothelial calcium-dependent NO synthase immunoreactivity was observed in poorly differentiated infiltrating duct carcinoma cells. In conclusion, expression of endothelial calcium-dependent NO synthase in human breast apocrine metaplasia may be of significance in view of the NO's vascular effects in benign breast disease.

**Keywords:** nitric oxide; apocrine metaplasia; breast cancer

Apocrine metaplasia or pink cell change of the breast, associated with cystic breast epithelium is characterised by high cylindrical cells with granular eosinophilic cytoplasm and luminal cytoplasmic projections (Bonser *et al.*, 1961).

For many years apocrine metaplastic cells have been regarded as having little or no significance in relation to malignant breast disease. However, based on the findings of several independent studies (Page *et al.*, 1978; Roberts *et al.*, 1984; Dixon *et al.*, 1985; Haagensen, 1986; Wellings *et al.*, 1987), it was concluded that metaplastic apocrine change reflects a significant epithelial unrest associated with carcinoma (Wellings *et al.*, 1987).

Since nitric oxide (NO), which is an inorganic free radical gas, synthesised by a family of isoenzymes called NO synthases (Ängard, 1994), is known to act not only as a vasorelaxant, but also as a cytostatic/cytotoxic mediator (Förstermann *et al.*, 1994), its role in tumour biology is under thorough investigation, but up to now remains poorly understood.

Thomsen *et al.* (1994) recently reported endothelial calcium-dependent NO synthase (eNOS) activity in human gynaecological neoplasms, and its presence seems to correlate inversely with the differentiation of the tumour. In contrast, the same investigators could not find any activity in breast tumour cells, as was demonstrated by immunohistochemistry, whereas tumour-infiltrating macrophages, endothelial and myoepithelial cells were immunoreactive with a polyclonal antiserum to NO synthase (Thomsen *et al.*, 1995).

As it was suggested that NO may not only cause tumour cell cytostasis/cytotoxicity but may also increase tumour blood flow (Andrade *et al.*, 1992) and promote angiogenesis (Jenkins *et al.*, 1995) we designed this study to determine if apocrine metaplastic change in benign breast disease is associated with the presence of immunohistochemically detectable eNOS.

### Materials and methods

### *Immunohistochemistry*

The staining reactions were performed on frozen sections of 30 samples of breast tissue. Of these samples, 21 were fibrocystic disease, four fibroadenoma, and five were poorly differentiated ductal carcinoma. For immunohistochemistry, a monoclonal anti-eNOS antibody was used at a concentration of 2.5 $\mu g\ ml^{-1}$ (Transduction Laboratories, Lexington, KY, USA). Specificity of the staining for eNOS was evident from its elimination by preabsorption with the eNOS peptide and the absence of staining with preimmune serum as was recently described (Dinerman *et al.*, 1994). For the immunodetection, a high-performance biotin-streptavidin detection system was used (Bio Genex, San Ramon, CA, USA). Vascular endothelial cells in the samples were used as the positive control; a non-immune serum was used for the negative control. For the chromogen reactions, aminoethylcarbazol, which forms a brown colour, was used with blocking reagents being included. The sections were finally counterstained with Mayer's haematoxylin and mounted with an aqueous medium.

An additional control to exclude cross-reactivity of the eNOS antibody with n (neuronal) NOS was recently described by performing eNOS and nNOS immunostaining on human cerebral arteries (Dinerman *et al.*, 1994). eNOS immunoreactivity was prominent in the endothelial cell layer of the middle cerebral artery but not in the adventitia, whereas nNOS immunoreactivity was confined to nerve fibres of the adventitia but not vascular endothelium (Dinerman *et al.*, 1994).

### *Histochemistry*

For NADPH-diaphorase stains the method by Hope and Vincent (1989) was slightly modified for titration and subsequent quantification of the NADPH-diaphorase activity. Five 10-$\mu$m-thick sections were cut from additional three frozen samples of apocrine metaplasia within a fibrocystic disease and were mounted on glass slides. Increasing NADPH-diaphorase activity was identified by incubating the slides with 50 mM Tris-buffered saline (pH 7.5) containing 2, 1, 0.5 or 0.25 mM NADPH (Sigma, St Louis, MO, USA), 0.5 mM nitroblue tetrazolium (NBT) and 0.2% Triton X-100 at 37°C for 30 min. Control sections were exposed to the staining solution without NADPH.

All the slides were randomised and coded and then examined and assessed for the immunohistochemical and histochemical staining independently by two observers (WT and KC).

Correspondence: W Tschugguel, University of Vienna, AKH, Department of Gynecology & Obstetrics, Division of Endocrinology and Sterility Treatment, Währinger Gürtel 18-20, EBO 05, A-1090 Vienna, Austria
Received 2 January 1996; revised 21 May 1996; accepted 31 May 1996

*Western blotting analysis*

To exclude eNOS antibody cross-reactivity with i (inducible) NOS, Western blot analysis using the anti-eNOS antibody with 15 $\mu$g total cell lysate from mouse macrophages (Transduction Laboratories), treated with interferon gamma (IFN-$\gamma$) and lipopolysaccharide (LPS) for 12 h, was performed. The lysate was heated at 100°C for 5 min. The whole tissue lysate, which contained 15 $\mu$g of protein, was subjected to sodium dodecyl sulphate-polyacrylamide gel electrophoresis (SDS-PAGE) (7.5% gradient). The separated proteins were electrophoretically transfered to membranes, then incubated with the eNOS antibody for 1 h. The bound antibody was detected using a chemoluminescent detection kit (ECL Western blotting detection system, Amersham, Arlington Heights, IL, USA), according to the manufacturer's instructions. No appropriately sized protein was detectable (data not shown).

### Results

We used a monoclonal antibody to eNOS to identify and localise it in our sections. Of the 25 benign breast disease tissue blocks studied, four specimens with fibroadenoma were negative for specific staining (results not shown), whereas the remaining 21 showed the following typical staining characteristics agreed upon by both the observers. Apocrine metaplastic change was consistently associated with the strongest intensity of staining (Figure 1) in all of the 21 specimens, and the reaction was predominantly localised

**Figure 1** Mammary cyst with immunostaining of apocrine metaplastic cells within a benign breast cyst (bar = 50 $\mu$m).

**Figure 2** Staining of endothelial cells in a fibrocystic breast disease (bar = 20 $\mu$m).

**Figure 3** Localisation of NO synthase in endothelial cells within an infiltrating duct carcinoma, poorly differentiated (bar = 20 $\mu$m).

**Figure 4** NADPH-diaphorase activity in apocrine metaplastic and adjacent endothelial cells within a benign breast cyst (bar = 20 $\mu$m), using (**a**) 1 mM NADPH and (**b**) 0.25 mM NADPH. (**c**) Lack of staining by incubation without NADPH.

within the cell cytoplasm. Flattened cyst epithelium showed no specific staining in all of the cases studied. Morphologically normal lobules, ducts, blunt ducts and adenosis also gave no immunoreactivity. Background staining was not visible in the majority of the cases, although non-specific artefacts that were interpreted as drying artefacts could be observed in some of the sections. In addition, immunolabelling of vascular endothelial cells was widespread throughout all tissue sections (Figure 2). The labelling of these cells was just as evident as that observed in apocrine metaplastic cells.

No immunolabelling was observed in breast tumour cells of poorly differentiated, infiltrating duct carcinomas (five specimens), whereas the endothelial cells of small tumour vessels and capillaries were intensely immunostained (Figure 3). A control section proved that there was no specific immunostaining in the absence of the eNOS antibody (results not shown).

To confirm the presence of NOS activity (Hope and Vincent, 1989) in apocrine metaplastic cells, NADPH–diaphorase stains with increasing concentrations of NADPH were done on frozen sections of fibrocystic disease of the breast. In this NADPH-dependent reaction, NBT is reduced to the water-insoluble dye, NBT formazan. The reaction does not distinguish among the various isoforms of NOS. Reaction product was identified in both apocrine metaplastic cells and endothelial cells at 2, 1 (Figure 4a) and 0.5 mM NADPH. At a concentration of 0.25 mM NADPH, reaction product was observed in apocrine metaplasia, but not in endothelium throughout all samples (Figure 4b).

### Discussion

In this study we have extended our previous observations, whereby we identified eNOS in several breast cancer cell lines (Zeillinger *et al.*, 1996). We have now mapped immunohistochemically the distribution of this NO synthase isoform using a monoclonal antibody for detection in frozen sections of benign breast disease and ductal carcinoma of the breast. Moreover, we used NADPH–diaphorase staining to confirm the presence of enzymatic NOS activity in apocrine metaplastic cells.

Our results indicate for the first time that eNOS is consistently demonstrable in histologically definable apocrine metaplastic cells found within benign breast disease. The specific eNOS immunostaining was also found to have a high regularity in vascular endothelial cells throughout all specimens, which is consistent with the fact that this NO synthase isoform was first detected in endothelial cells. For quantification of biochemical enzyme activity NADPH–diaphorase staining was compared between apocrine metaplastic and endothelial cells by incubating additional sections with increasing concentrations of NADPH. Both cell types were particularly prominent sites of eNOS protein. Whereas NADPH–diaphorase activity in endothelial cells was predictable, the stronger staining pattern in apocrine metaplastic cells compared with endothelial cells was unexpected. This might reflect either stronger eNOS activity or additional activity in apocrine metaplasia as a result of co-expression of the other NOS isoenzymes, but remains to be explored further.

Haagensen (1991) suggested three possible roles for apocrine metaplasia. First, apocrine metaplasia may be a precursor to malignant transformation. The second possibility is that it reflects a response to the same stimulus that can also induce carcinoma. Finally, these changes may have a higher propensity for malignant changes.

Apocrine metaplastic cells are also associated with a strong immunostaining for prolactin (Kumar *et al.*, 1987). Moreover, this hormone was also found with a high regularity in breast cancer specimens and, thus, led to the hypothesis that metaplastic cells may be of significance in view of the hormone's known growth-stimulating effect on the breast epithelium (Kumar *et al.*, 1987).

In gynaecological cancer tissue (ovarian, endometrial and mixed mesodermal tumours), the presence of eNOS was shown to correlate inversely with the differentiation of the tumour (Thomsen *et al.*, 1994). Its activity was significantly higher in tissue explants of poorly differentiated compared with moderately differentiated ovarian cancers (Thomsen *et al.*, 1994). Unexpectedly, the same investigators reported lack of any NO synthase immunolabelling in breast tumour cells, whereas tumour-infiltrating macrophages, myoepithelial cells and endothelial cells of tumour vasculature were intensely immunostained (Thomsen *et al.*, 1995). We have already demonstrated that several human breast cancer cell lines express eNOS mRNA, although in much lower amounts compared with cultured human umbilical vein endothelial cells (Zeillinger *et al.*, 1996). This eNOS expression strongly correlated with the oestrogen receptor status of these lines. Cell lines that did not express the oestrogen receptor mRNA did not express NO synthase mRNA. Thus, it was hypothesised that oestradiol–perhaps in connection with an up-regulation of the oestrogen receptor–may be a strong enhancer for NO release in breast tumour cells (Zeillinger *et al.*, 1996). The discrepancy between both studies (Zeillinger *et al.*, 1996; Thomsen *et al.*, 1995) could be explained methodologically: it may be a result of different sensitivity of the detection method by polymerase chain reaction on the one hand (Zeillinger *et al.*, 1995), and immunohistochemistry (Thomsen *et al.*, 1995) on the other hand. This implies that breast cancer cells express the eNOS protein at a much lower level than in apocrine metaplastic cells; levels that would probably be irrelevant in a tumour in which there are other abundant sources of NO generation, such as macrophages and endothelial cells.

In addition, results from three specimens of invasive ductal carcinoma that were immunohistochemically stained with a monoclonal eNOS antibody (Figure 3) were consistent with those obtained by Thomsen *et al.* (1995).

However, one can hypothesise that the expression of NO synthase in tumour-infiltrating macrophages would contribute to the cytotoxic effect of NO on tumour cells, whereas expression in vascular endothelial and myoepithelial cells may also increase tumour blood flow and promote angiogenesis (Weidner *et al.*, 1992). Thomsen *et al.* (1995) concluded that a balance in favour of the vascular effects may explain the positive correlation between NO biosynthesis and grade of malignancy. Thus, it was suggested that NO may have a dual pro- and anti-tumour action (Jenkins *et al.*, 1995). By considering NO's vascular effects and the proposal that the free radical NO may also act as a mutagen (Wink *et al.*, 1991; Zeillinger *et al.*, 1996), we conducted our study to examine its expression in apocrine metaplastic cells that demonstrate a strong association with an increased breast cancer risk (Page *et al.*, 1978; Roberts *et al.*, 1984; Wellings *et al.*, 1987).

eNOS immunolabelling was detectable in apocrine metaplasia, with intense staining similar to that of endothelial cells (Figures 1 and 2). If this apocrine metaplastic cell is viewed as a preneoplastic cell, a morphogenetical relationship between apocrine metaplastic cells and neoplastic breast cancer cells could be suggested.

In conclusion, human breast apocrine metaplasia that was shown to be associated with an increased breast cancer risk expresses endothelial calcium-dependent NO synthase. This may be of significance in view of the NO's vascular effects in benign breast disease.

**Acknowledgement**
Supported by grant P01437–MED from the Austrian Science Foundation.

### References

ANDRADE SP, HART IR AND PIPER PJ. (1992). Inhibitors of nitric oxide synthase selectively reduce flow in tumor-associated neovasculature. *Br. J. Pharmacol.*, **107**, 1092–1097.

ÄNGARD E. (1994). Nitric oxide: mediator, murderer, and medicine. *Lancet*, **343**, 1199–1206.

BONSER GM, DOSSETT JA AND JULL JW. (1961). *Human and Experimental Breast Cancer*. Pitman Medical: London.

DINERMAN JL, DAWSON TM, SCHELL MJ, SNOWMAN A AND SNYDER SH. (1994). Endothelial nitric oxide synthase localized to hippocampal pyramidal cells: implications for synaptic plasticity. *Proc. Natl Acad. Sci. USA*, **91**, 4214–4218.

DIXON JM, LUMSDEN AB AND MILLER WR. (1985). The relationship of cyst type to risk factor for breast cancer and the subsequent development of breast cancer in patients with breast cystic disease. *Eur. J. Cancer Clin. Oncol.*, **21**, 1047–1050.

FÖRSTERMANN U, CLOSS EI, POLLOCK JS, NAKANE M, SCHWARZ P, GATH I AND KLEINERT H. (1994). Nitric oxide synthase isozymes: characterization, purification, molecular cloning, and functions. *Hypertension*, **23**, 1121–1131.

HAAGENSEN CD. (1986). *Diseases of the Breast*. p.95. WB Saunders Co.: Philadelphia.

HAAGENSEN DE Jr. (1991). Is cystic disease related to cancer? *Am. J. Surg. Pathol.*, **15**, 687–694.

HOPE BT AND VINCENT SR. (1989). Histochemical characterization of neuronal NADPH-diaphorase. *J. Histochem. Cytochem.*, **37**, 653–661.

JENKINS DC, CHARLES IG, THOMSEN LL, MOSS DW, HOLMES LS, BAYLIS SA, RHODES P, WESTMORE K, EMSON PC AND MONCADA S. (1995). Roles of nitric oxide in tumor growth. *Proc. Natl Acad. Sci. USA*, **92**, 4392–4396.

KUMAR S, MANSEL RE AND JASANI B. (1987). Presence and possible significance of immunohistochemically demonstrable prolactin in breast apocrine metaplasia. *Br. J. Cancer*, **55**, 307–309.

PE DL, ZWAGG RV, ROGERS LW, WILLIAMS LT, WALKER WE AND HARTMAN WH. (1978). Relation between component parts of fibrocystic desease complex and breast cancer. *J. Natl Cancer Inst.*, **61**, 1055–1063.

ROBERTS MM, JONES V, ELTON RA, FORTT RW, WILLIAMS S AND GRAVELLE IH. (1984). risk of breast cancer in women with history of benign disease of the breast. *Br. Med. J.*, **288**, 275–278.

THOMSEN LL, LAWTON FG, KNOWLES RG, BEESLEY JE, RIVEROS-MORENO V AND MONCADA S. (1994). Nitric oxide synthase activity in human gynecological cancer. *Cancer Res.*, **54**, 1352–1354.

THOMSEN LL, MILES DW, HAPPERFIELD L, BOBROW LG, KNOWLES RG AND MONCADA S. (1995). Nitric oxide synthase activity in human breast cancer. *Br. J. Cancer*, **72**, 41–44.

WEIDNER N, FOLKMAN J, POZZA F, PIERANTONIO B, ALLRED EN, MOORE DH, MELI S AND GASPARINI G. (1992). Tumor angiogenesis: a new significant and independent prognostic indicator in early-stage breast carcinoma. *J. Natl Cancer Inst.*, **84**, 1875–1887.

WELLINGS SR AND ALPERS CE. (1987). Apocrine cystic metaplasia: Subgross pathology and prevalence in cancer-associated versus random autopsy breasts. *Hum. Pathol.*, **18**, 381–386.

WINK DA, KASPRZAK KS, MARAGOS CM, ELESPURU RK, MISRA J, DUNAMS TM, CEBULA TA, KOCH WH, ANDREWS AW, ALLEN JS AND KEEFER LK. (1991). DNA deaminating ability and genotoxicity of nitric oxide and its progenitors. *Science*, **254**, 1001–1003.

ZEILLINGER R, TANTSCHER E, SCHNEEBERGER C, TSCHUGGUEL W, EDER S, SLIUTZ G AND HUBER JC. (1996). Simultaneous expression of nitric oxide synthase and estrogen receptor in human breast cancer cell lines. *Breast Cancer Res. Treat.*, **40**, 205–207.